# Phthalate Diesters and Their Metabolites in Human Breast Milk, Blood or Serum, and Urine as Biomarkers of Exposure in Vulnerable Populations

**DOI:** 10.1289/ehp.10788

**Published:** 2007-12-21

**Authors:** Johan Högberg, Annika Hanberg, Marika Berglund, Staffan Skerfving, Mikael Remberger, Antonia M. Calafat, Agneta Falk Filipsson, Bo Jansson, Niklas Johansson, Malin Appelgren, Helen Håkansson

**Affiliations:** 1 Institute of Environmental Medicine, Karolinska Institutet, Stockholm, Sweden; 2 Section of Occupational and Environmental Medicine, University Hospital of Lund, Sweden; 3 IVL Swedish Environmental Research Institute Ltd., Stockholm, Sweden; 4 Division of Laboratory Sciences, National Centre for Environmental Health, Centers for Disease Control and Prevention, Atlanta, Georgia, USA; 5 Department of Applied Environmental Sciences, Stockholm University, Stockholm, Sweden; 6 Swedish Environmental Protection Agency, Stockholm, Sweden

**Keywords:** biomonitoring, blood, breast milk, metabolites, perinatal, phthalates, urine

## Abstract

**Background:**

Phthalates may pose a risk for perinatal developmental effects. An important question relates to the choice of suitable biological matrices for assessing exposure during this period.

**Objectives:**

This study was designed to measure the concentrations of phthalate diesters or their metabolites in breast milk, blood or serum, and urine and to evaluate their suitability for assessing perinatal exposure to phthalates.

**Methods:**

In 2001, 2–3 weeks after delivery, 42 Swedish primipara provided breast milk, blood, and urine samples at home. Special care was taken to minimize contamination with phthalates (e.g., use of a special breast milk pump, heat treatment of glassware and needles, addition of phosphoric acid).

**Results:**

Phthalate diesters and metabolites in milk and blood or serum, if detected, were present at concentrations close to the limit of detection. By contrast, most phthalate metabolites were detectable in urine at concentrations comparable to those from the general population in the United States and in Germany. No correlations existed between urine concentrations and those found in milk or blood/serum for single phthalate metabolites. Our data are at odds with a previous study documenting frequent detection and comparatively high concentrations of phthalate metabolites in Finnish and Danish mothers’ milk.

**Conclusions:**

Concentrations of phthalate metabolites in urine are more informative than those in milk or serum. Furthermore, collection of milk or blood may be associated with discomfort and potential technical problems such as contamination (unless oxidative metabolites are measured). Although urine is a suitable matrix for health-related phthalate monitoring, urinary concentrations in nursing mothers cannot be used to estimate exposure to phthalates through milk ingestion by breast-fed infants.

Phthalates are used in large quantities as softeners in many plastic products, paint, glue, putty, pharmaceutical products and cosmetics (Agency for Toxic Substances and Disease Registry 2001, 2002; Schettler 2006). About 5,000–6,000 tons of phthalates, including di(2-ethylhexyl) phthalate (DEHP), are used per year in Sweden. Typically, phthalates are not chemically bound to the product matrix and may thus migrate and permit extensive exposure. People may be exposed in the work environment, via food from plastic containers and via inhalation of dust in domestic environments (Wormuth et al. 2006). Dermal exposure via clothes and cosmetics may also occur. Small population groups may be exposed via medical equipment—for example, to DEHP migrating from plastic tubing used for treatment of premature infants (Calafat et al. 2004a; Weuve et al. 2006).

Numerous studies have reported reproductive effects, including reduced sperm production and shortened anogenital distance (AGD) in laboratory animals, and concern exists that phthalates may induce antiandrogenic and/or proestrogenic effects in humans (Andrade et al. 2006; Borch et al. 2006; Fabjan et al. 2006; Gray et al. 2006; Hauser 2006; Hauser et al. 2006; Pflieger-Bruss et al. 2004). Developmental defects in male rat pups, similar to those seen in humans in a syndrome termed testicular dysgenesis syndrome (TDS), have been documented after dosing pregnant dams with di-*n*-butyl phthalate (DBP), DEHP, or butyl benzyl phthalate (BBzP) (Sharpe 2005). Signaling pathways affected by phthalates are currently under intense study (Ge et al. 2007; Hallmark et al. 2007; Howdeshell et al. 2007; Lahousse et al. 2006; Liu et al. 2005; Mahood et al. 2006; Sharpe 2006).

Phthalate diesters are metabolized, and most studies of human exposure report concentrations of phthalate metabolites in urine. In the body, hydrolytic monoester metabolites can undergo phase 1 (e.g., oxidation) and phase 2 (e.g., glucuronidation) metabolism (Koch et al. 2003b). In biological samples, such as blood or serum, milk, or other complex biological samples, contaminating phthalate diesters may be hydrolyzed by esterases to their respective hydrolytic monoesters during sampling, storage, and analysis (Kato et al. 2004). However, oxidized monoester metabolites cannot easily be ascribed to contamination (Koch et al. 2003b, 2004, 2005, 2006b).

Recent U.S. studies summarize data from > 2,500 individuals participating in the National Health and Nutrition Examination Survey (NHANES) [Centers for Disease Control and Prevention (CDC) 2005; Silva et al. 2004a]. In general, higher concentrations of urinary metabolites were found in women than in men. This pattern was also seen in a German study (Koch et al. 2003b), and other studies show ethnic differences (CDC 2005; Silva et al. 2004a).

The toxicologic profile of phthalates and perhaps a higher prevalence of exposure among women suggest that pregnant women, fetuses, and newborns could be highly sensitive risk groups. In the present study, performed in 2001 at the request of the Swedish Environmental Protection Agency, we measured several common phthalates and their metabolites in human milk, blood or serum, and urine. The main objectives of this study were to evaluate whether multimatrix biomonitoring of phthalates in women of child-bearing ages is feasible, and to identify the most suitable biological matrix for health-related environmental monitoring of risk groups in the general population.

## Methods

### Study group

Of about 130 consecutive Swedish women approached in connection with a normal delivery at Lund University Hospital in southern Sweden, 68 were willing to participate in the study. Of those, 42 (median age, 29; range, 23–39 years) participated; the dropout was caused by logistic problems in sampling, postdelivery tiredness, or scarcity of breast milk. Two women were smokers, 35 were nonsmokers, and five were ex-smokers.

The project was approved by the Ethical Committee at Lund University and by the Institutional Review Board at the CDC. Participants gave written informed consent.

### Interview

The mothers were visited 4–5 times in their homes by a nurse. At one of the visits, the mothers were interviewed, and at the others only sampling took place. The mothers were interviewed about factors that might have contributed to their exposure to phthalates: the delivery (including medication), body weights of mother and baby, the mother’s height, age, education, family, occupation, place of residence, renovation of the home, car, exercise, lifestyle habits (use of coffee, tea, cigarettes, snuff), use and brands of cosmetic and hygiene products (perfume, antiperspirant, skin lotion, toothpaste, detergents), health, and medication.

Six (14%) mothers reported a chronic disease (allergy, eczema, treated hypothyroidism). Nine (21%) mothers had suffered some kind of pregnancy problems. Twenty-seven (64%) of the mothers had > 3 years of university education, five (12%) had 1–3 years of university education, eight (19%) had 3–4 years of high school education, and the remaining two mothers (5%) had 1–2 years of high school education. Twenty-four mothers lived in a private house and 18 in apartments; 26 of the mothers reported that their home had been renovated since they moved there. The babies were 25 girls and 17 boys. Their birth weight (mean ± SD) was 3,586 ± 450 g.

### Sampling

#### Breast milk

All 42 mothers gave milk samples. At the time of breast milk sampling, the babies were 14–20 days of age (median, 17 days). The mothers were instructed not to use skin care products before sampling.

The milk samples were collected by using a pump made of polycarbonate. The pump consisted of a piston in cylinder housing (Amningspump; Artaplast, Tyresö, Sweden). The rubber seal of the piston of the commercial pump was found to contain high concentrations of DEHP. Even when the seal was replaced by a phthalate-free one, high levels of DEHP appeared in the blank samples. Hence, new pumps with fluoroelastomer (black Viton; Norton Verneret, Cavaillon, France) tubing seal were used. The pump was rinsed with ethanol before use. The milk was transferred to preheated (400°C for 2 hr) laboratory glass bottles with glass stoppers. The samples were stored in a refrigerator (4°C) for a couple of hours. After collection of 50–100 mL milk, phosphoric acid was added and the samples were stored in a freezer (−80°C) until transferred to the laboratory at the Swedish Environmental Research Institute Ltd. (IVL) and analyzed.

#### Blood

Thirty-six of the mothers gave blood samples, collected 1 week after milk sampling. Blood was taken with a stainless steel needle from a cubital vein and allowed to drop into preheated glass test tubes. Five milliliters of blood were mixed with 10,000 IE of heparin. Phosphoric acid was added and the tube openings were covered by clean aluminium foil to protect the sample from contacts with the polytetraethylenefluoride screw caps. The samples were stored in a freezer (−20°C) until transferred to IVL and analyzed. Further, untreated blood was centrifuged. Serum was collected by using phthalate-free tubing and a heated pipette, mixed with phosphoric acid, and stored in a freezer.

#### Urine

Thirty-eight of the mothers provided urine samples at the time of blood sampling. About 100 mL urine was collected in a heated glass beaker, and 5 mL was frozen (−80°C) in a tube and transferred to the laboratory at the CDC.

#### Additional measures to control for contamination

Chemicals added to the samples, heparin and ethylenediaminetetraacetic acid, were checked by gas chromatography–mass spectrometry (GC-MS) before use. Nitrile plastic gloves, containing negligible amounts of phthalates (assessed by GC-MS) were used in this study when collecting body fluids. Milk and blood samples were acidified with phosphoric acid (1 M; 125 μL/mL) about 1 hr after the collection (Calafat et al. 2004b; David and Sandra 2001) to avoid enzymatic hydrolysis of the phthalate diesters. The samples were stored in a freezer at the University Hospital of Lund.

### Measurement of phthalate diesters in blood and milk

The phthalate diesters were measured at IVL. Because of the ubiquitous presence of these compounds, all solvents (Rathburn Chemical Ltd., Peeblesshire, Scotland) used were checked, and the cleaning procedure for equipment of glass and metal (and inorganic salts) included heating at 400°C for 4 hr. At least two blanks were analyzed along with each batch of samples to monitor background concentrations of phthalate diesters.

The blood samples (10 mL) were thawed and diluted with ultrapure water. Surrogate standards were added and the sample was extracted twice with 5mL hexane:MTBE (1:1) and once with 3 mL hexane. The extract was concentrated and cleaned up on an amino propylene column (International Sorbent Technology, Ltd., Tucson, AZ, USA).

The milk samples (10 mL) were thawed and extracted according to a method described earlier (David and Sandra 2001). Briefly, the sample was extracted with pentane:acetone and hexane:MTBE after addition of sodium chloride and ultrapure water. The acetone was washed away with water, and the extract was subjected to cleanup using gel permeation chromatography [5 mL/min pentane:MTBE (1:1), PL-gel column, 300 × 25 mm; 10 μm, 50 Å, Polymer Laboratories Varian, Inc., Amherst, MA, USA] and the amino propylene column.

We analyzed the extracts using GC-MS with a mass selective detector in selected ion monitoring mode (Agilent 6890N; Agilent Technologies Inc., Santa Clara, CA, USA). The analytes were identified by their *a*) characteristic retention time, *b*) one quantification ion, and, in most cases, *c*) one confirmation ion to increase specificity. The calibration standard was a certified standard mixture of six phthalate esters (Ultra Scientific; J.T. Baker, Pillipsburgh, NJ, USA). The mixture contained dimethyl phthalate, diethyl phthalate (DEP), DBP, BBzP, DEHP, and di-*n*-octyl phthalate (DOP). The commercial isomeric mixtures di-isononyl phthalate (DINP) and di-isodecyl phthalate (DIDP) were kindly supplied bv Perstorp Oxo AB (Stenungsund, Sweden). The limits of detection (LODs) ranged from 0.1 to 3.0 ng/mL. Quantification was based on the internal standards, and the reported concentrations were corrected for the recovery of those standards.

The recovery rate according to surrogate standards was 66 ± 11% for the milk samples and 58 ± 15% blood samples. The results of the spiked control samples, analyzed in parallel with the samples, are presented in Table 1. All reported values were recovery corrected according to the surrogate standards. The precision was determined to 20% at the concentrations detected in the samples. A calculation of the uncertainty in the measurement (Ellison et al. 2000) results in an uncertainty of 19% in the measurement.

### Measurement of phthalate metabolites in serum, milk, and urine

Phthalate metabolites were measured in urine, breast milk, and serum at the CDC. The analytic methods involved the enzymatic deconjugation of the phthalate metabolites from their glucuronidated form, automated solid-phase extraction, separation with high performance liquid chromatography, and detection by isotope-dilution tandem mass spectrometry (Calafat et al. 2004b; Kato et al. 2004; Silva et al. 2004b). The LODs were in the low nanogram per milliter range; 1 mL of sample was used for the analysis. Each analytic run consisted of 50 samples [2 quality control (QC) materials of high concentration, 2 QC materials of low concentration, 5 reagent blanks, and 41 unknown samples]. We analyzed the QC samples along with unknown samples to monitor for accuracy and precision. QC samples were evaluated according to modified Westgard statistical probability rules.

### Statistical analysis

For univariate associations between the continuous variables, we used the Spearman correlation test (*r*_s_ = Spearman correlation coefficient) from SigmaStat for Windows (version 3.5; Systat Software Inc. San Jose, CA, USA). For concentrations < LOD, unless stated otherwise, we imputed a value equal to half the LOD for the calculations. *p*-Values < 0.05 were considered statistically significant.

We used multivariate modeling and analysis to explore possible correlations between concentrations of individual phthalates, their metabolites, and interview variables. We used principal component and partial least squares (PLS) analyses as performed by the statistical package Simca-P 10.0 (Umetrics AB, Umeå, Sweden). The PLS method was used as described previously (Wold et al. 1984) and is suited for identification and evaluation of systematic variation in complex data sets.

## Results

Tables 2–5 summarize the concentrations of phthalate diesters and phthalate metabolites measured in several biological media (blood or serum, breast milk, urine), as well as the number of samples giving results above the LOD. Most blood or serum and milk samples had phthalate and phthalate metabolite concentrations below the LOD, but all urine samples had detectable concentrations of most metabolites. No blood or milk sample contained detectable concentrations of DIDP or DINP.

The urinary concentrations of oxidized metabolites of DEHP [mono(2-ethyl-5-oxohexyl) phthalate (mEOHP; *r*_s_ = 0.30, *p* = 0.06) and mono(2-ethyl-5-hydroxyhexyl) phthalate (mEHHP; *r*_s_ = 0.47, *p* = 0.003)] correlated with the concentrations of the hydrolytic monoester metabolite mono(2-ethylhexyl) phthalate (mEHP), suggesting that these three metabolites reflect DEHP exposure. Although the concentrations of mEHHP and mEOHP in urine were higher than the concentrations of mEHP, these oxidative metabolites were not detected in serum or milk. By contrast, mEHP was detected in a small percentage of milk and serum samples (Tables 2 and 3). The range of urine concentrations was smaller for the DEHP metabolites than for mono-*n*-butyl phthalate (mBP) and monoethyl phthalate (mEP).

Except for one sample exhibiting a high concentration of DEHP in blood (129 ng/mL), most blood concentrations were close to or below the LOD. The high blood concentration of DEHP was not matched by a high concentration of DEHP metabolites in serum or urine from the same woman, suggesting that contamination may have affected this analysis. With this exception, our blood DEHP and serum mEHP results agree with previously reported serum concentrations of DEHP (< 14 ng/mL) and mEHP (< 5 ng/mL) from four individuals (Takatori et al. 2004).

Concentrations of phthalates and phthalate metabolites in milk (*n* = 42) were close to or below the LOD, except for one sample exhibiting a high concentration (305 ng/mL) of DEHP. The woman with high DEHP in milk was not the one with high DEHP in blood, and her mEHP concentration in milk was low (unfortunately, we had no blood or urine data from this woman). In general, the milk concentrations of metabolites were lower than the corresponding parent compounds concentrations in milk. We did not observe significant correlations between parent compounds and the corresponding metabolites in milk (DEHP–mEHP: *r*_s_ = 0.023, *n* = 42; DBP–mBP: *r*_s_ = −0.12, *n* = 42). For DBP, the lack of correlation may be attributable to the low number of samples with concentrations above the LOD. If only samples above the LOD (*n* = 12) were included in the analysis, we observed a negative significant correlation between DBP and mBP in milk (*r*_s_ = −0.63; *p* = 0.024).

In a search for correlations between concentrations in different matrices, we drew several plots, but no obvious patterns could be discerned. However, there was an apparent correlation between urine mEP and serum mEP (7 values > LOD) (*r*_s_ = 0.48, *p* < 0.003, *n* = 36), but no correlation with blood DEP (29 values > LOD), so the biological significance of this correlation is uncertain. After removal of an apparent outlier, there was still a significant correlation (*r*_s_ = 0.41, *p* = 0.014, *n* = 35). It is possible that this correlation reflects the urinary excretion of mostly unconjugated mEP, whereas variable conjugation rates (due to polymorphisms in the phase 2 glucuronidation reaction enzyme, uridine 5′-diphosphate glucoronosyltransferase) may have eroded correlations for other monoester metabolites that are generally excreted in their conjugated form. Women having detectable milk concentrations of mEHP exhibited a slightly elevated, although not statistically significant, ratio between mEHP/(mEOHP + mEHHP) concentrations in urine.

We performed a multivariate analysis using the analytic results and the individual data collected in the interviews. The complete data set and selected subsets did not reveal any significant correlations between concentrations of individual phthalate diesters, their metabolites, or any of the exposure variables recorded, except those described above for urinary DEHP metabolites and for mEP in urine and serum. In several models, we observed weak positive associations between age and the concentrations of a few metabolites in milk and urine.

## Discussion

The present study population is small, though carefully recruited. It might be argued that the study group was not representative for the average Swedish woman. The participants were selected from a three times larger group of potential participants, and selected individuals had a relatively high level of education. This may partially relate to the fact that they lived in a small university town and might have been more interested than average women in the study results. However, we do not think that this selection has seriously biased our results. Also, we made great efforts to avoid contamination with and degradation of phthalates. This included the use of a special milk pump, heat treatment of glassware and needles, and addition of phosphoric acid.

The urinary concentrations of phthalate metabolites in the Swedish mothers studied here are in the ranges previously reported for other population groups living in Germany and the United States (CDC 2005; Koch et al. 2003a; Silva et al. 2004a; Swan et al. 2005) (Table 6). The strong correlations seen between the urinary concentrations of the three DEHP metabolites were expected and argue against contamination in the urine samples. The distribution of the various metabolites may thus reflect common exposures of humans. In a group of 85 Germans, the calculated daily intake of DEHP (based on urinary metabolite concentrations, shown here in Table 6) exceeded the European tolerable daily intake value, based on reproductive toxicity [CSTEE (European Scientific Committee on Toxicity, Ecotoxicity and Environment) 1998], for 12% of the individuals (Koch et al. 2003a). Similarly, on the basis of similar urinary data, Koch et al. (2006a) concluded that about one third of 2- to 4-year-old German children had an intake of DBP that exceeded the tolerable daily intake.

Somewhat worrisome perhaps are our data on the metabolites of DBP, DEHP, and BBzP, which have been found to produce a TDS-like syndrome in rats (Hallmark et al. 2007; McKinnell et al. 2005; Sharpe 2005). The metabolites of these phthalates were detected (Table 6) at slightly higher median urinary concentrations among this group of Swedish mothers than those found in one study correlating phthalate exposure in the United States to a shortened AGD (Swan et al. 2005). Toxic effects of mixtures of antiandrogens may exhibit additivity (Howdeshell et al. 2007; Metzdorff et al. 2007), and the sum of medians for the DBP, DEHP, and BBzP metabolites were 378 nmoles/L in the Swedish samples and 182 nmoles/L in the U.S. samples. However, the sum of all medians in Table 6 was higher in the U.S. samples (861 nmoles/L) than in the Swedish samples (646 nmoles/L). Only concentrations of mEP, mBP, monobenzyl phthalate (mBzP), and mono-iso-butyl phthalate (miBP) correlated with shortened AGD in the U.S. human study (Swan et al. 2005), and their sums of medians were 759 nmoles/L in the U.S. samples and 512 nmoles/L in the Swedish samples. However, the fact that prenatal/late pregnancy (the U.S. study) and postnatal (the Swedish study) samples were used for these analyses hampers the comparison because pregnancy alters pharmacokinetic parameters (Anderson 2005).

The blood and serum data are more difficult to interpret. We observed no obvious correlations between these concentrations and concentrations of the metabolites in urine, even for the oxidized metabolites, which should not have been affected by potential contamination (Kato et al. 2004). The urine and blood samples were collected up to 1 week after the milk samples, but urine concentrations are quite reproducible from day to day (Hoppin et al. 2002), so variability in urinary concentrations can hardly explain the lack of correlation. Therefore, it seems that having many serum concentrations below the LOD and the fact that the methodology used did not permit accurate analysis of prevailing low concentrations were important factors. Other factors that may erode correlations include hydrolysis of phthalate diesters introduced as contaminants during handling of the samples. Even dust in ambient air might be a factor of importance that is hard to control for in a domestic environment and under conditions employed to collect the samples in our study. Thus, considering the limitations in sensitivity of the analytic procedures, the potential contamination issues, as well as the discomfort associated with the blood sampling procedure, blood or serum sampling cannot be recommended for surveying phthalate exposure in the general population. Even surveying small targeted groups of susceptible individuals by using blood or serum samples might turn out to be much more complicated than using urine.

Collecting human milk samples was laborious, time consuming, and associated with potential contamination problems. It also required special sampling equipment and procedures. Furthermore, the milk samples obviously contained too low concentrations of phthalates and metabolites to permit a more comprehensive and detailed discussion of actual concentrations in this matrix. Despite the time difference in sampling urine and milk, our data do not support the notion that the mothers’ urinary concentrations can be used for assessing exposure through breast milk. However, if correlations do exist between urine and milk concentrations, and can be documented, the use of urine samples would greatly facilitate the study of exposure of breast-fed children.

Some data are available on levels of phthalates in human milk. Infant exposure to DEHP or mEHP via milk has been reviewed (Latini et al. 2004). Furthermore, analytic methods for measuring several phthalate metabolites in milk are available (Main et al. 2006; Mortensen et al. 2005), and data from a small number of pooled samples (Calafat et al. 2004b) and from two Danish/Finnish studies involving the analysis of individual human samples exist (Main et al. 2006; Mortensen et al. 2005). The recent Danish/Finnish studies (Main et al. 2006; Mortensen et al. 2005) report higher concentrations of some phthalate metabolites in human milk than we found. Specifically, the median concentrations for mBP were 4.3 and 12 μg/L, for mBzP were 0.9 and 1.3 μg/L, for mEHP were 9.5 and 13 μg/L, and for mEP were 0.93 and 0.97 μg/L, in Denmark and Finland respectively (Main et al. 2006). Considering the similarities in lifestyle and demographics between Sweden and the other two Nordic countries, these differences are surprising. Only a few of the Swedish samples exhibited values above the LOD (~ 1.0 ng/mL) for these metabolites (Table 2), although several of the concentrations reported by Main et al. (2006) should have been possible to detect with our analytic procedure. It has been speculated that contaminations may have influenced the results (Kavlock et al. 2006), and our data tend to support this. Also, in a recent review (Frederiksen et al. 2007), the same concentration figures are presented in nanograms per liter and not in nanograms per milliliter. In addition, the Danish/Finish samples were collected prospectively and earlier (1997–2001) than our samples, and temporal concentration trends of phthalates in the three Nordic countries are unknown. A closer analysis of factors that might explain these observed differences is thus urgent.

In conclusion, the results of this study indicate that sampling of breast milk or blood and analysis for phthalates is not a straightforward approach that can be recommended for surveillance of infants’ exposure to phthalates. Samples might easily be contaminated with the ubiquitous phthalate diesters. Our data also indicate that the analytic methods used, having LODs generally in the low parts per billion, were not sensitive enough for adequate quantification of common phthalates and their metabolites in human blood, serum, or milk in the Swedish population. In contrast, measuring phthalate metabolites in urine gives more informative results. The metabolite urinary concentrations in this group of Swedish women were similar to those reported among German and U.S. populations. However, it is evident that this study cannot answer the question whether analysis of maternal urine can be used to estimate exposure to phthalates through milk ingestion by breast-fed children. Additional research to address phthalate exposure in Swedish populations and in relation to AGD and other TDS-related end points is warranted.

## Figures and Tables

**Table 1:** Relative recovery (% of added amount) of phthalates spiked in milk and blood samples.

	DEP	DBP	BBzP	DEHP	DOP
Milk	104	86	88	97	77
Blood	95	98	108	103	100

The results are adjusted according to the surrogate standard.

**Table 2:** Concentrations of phthalates and their metabolites in milk (ng/mL).

	Parent compounds					Metabolites				
Milk	DEP	DBP	BBzP	DEHP	DOP	mBP	mBzP	mEHP	mEP	miBP
No. of samples	42	42	42	42	42	42	42	42	42	42
No. > LOD	8	12	41	39	10	11	3	16	1	2
Minimum	0.22	1.5	0.06	0.45	0.24	0.54	0.50	0.49	0.50	0.52
Maximum	1.45	20	4.4	305	11	5.7	4.4	6.5	2.5	2.1
Median	0.22	1.5	0.49	9.0	0.24	0.54	0.50	0.49		
75th percentile	0.22	3.1	0.92	13	0.24	1.3	0.50	1.7	0.50	0.52
Mean	0.30	2.8	0.75	17	1.1	1.2	0.64	1.3		
SD	0.24	3.4	0.80	47	2.3	1.3	0.63	1.3		
LOD	0.44	3.0	0.12	0.90	0.47	1.1	1.0	0.98	1.0	1.0

Values < LOD were set to LOD/2. The DEHP oxidative metabolites, MEHHP and MEOHP, were not detected in any of the samples analyzed.

**Table 3:** Concentrations of phthalates and metabolites in blood and serum (ng/mL).

	Blood parent compounds					Serum metabolites			
Blood and serum	DEP	DBP	BBzP	DEHP	DOP	mBP	mEHP	mEP	miBP
No. of samples	36	36	36	36	36	36	36	36	36
No. > LOD	29	25	29	17	7	17	6	7	3
Minimum	0.066	0.21	0.050	0.50	0.70	0.54	0.49	0.50	0.50
Maximum	1.1	9.1	1.4	129	10.0	20	4.5	14	11
Median	0.24	0.78	0.25	0.50	0.70	0.54	0.49	0.50	0.50
75th percentile	0.37	1.3	0.36	2.7	0.70	1.9	0.49	0.50	0.50
Mean	0.31	1.2	0.29	5.9	1.5	1.8	0.77	1.2	0.87
SD	0.26	1.6	0.27	21	2.1	3.3	0.80	2.3	1.8
LOD	0.13	0.43	0.10	1.0	1.4	1.1	0.98	1.0	1.0

Values < LOD were set to LOD/2. The DEHP oxidative metabolites, MEHHP and MEOHP, were not detected in any of the samples analyzed.

**Table 4:** Concentrations of phthalate metabolites in urine (ng/mL).

Urine	mBP	mBzP	mCPP	mEHHP	mEHP	mEOHP	mEP	mMP	miBP
No. of samples	38	38	38	38	38	38	38	38	38
No. > LOD	38	38	25	38	38	37	37	20	34
Minimum	5.1	2.2	0.5	1.4	2.9	0.54	0.50	0.50	0.52
Maximum	198	38	9.1	126	57	83	761	15	130
Median	46	13	1.5	15	9	11	35	1.2	16
75th percentile	68	20	2.5	29	17	24	80	2.8	30
Mean	53	16	1.9	25	13	19	84	2.3	21
SD	45	10	1.7	27	10	19	141	3.0	24
LOD	1.1	1.0	1.0	0.95	0.98	1.1	1.0	1.0	1.0

Abbreviations: mCPP, mono(3-carboxypropyl) phthalate; mMP, monomethyl phthalate. Values < LOD were set to LOD/2.

**Table 5:** Concentrations of phthalate metabolites in urine (μg/g creatinine).

Urine	mBP	mBzP	mCPP	mEHHP	mEHP	mEOHP	mEP	mMP	miBP
No. of samples	38	38	38	38	38	38	38	38	38
No. > LOD	38	38	25	38	38	37	37	20	34
Minimum	18	4.5	0.5	5.1	4.6	3.0	5.5	0.3	1.1
Maximum	191	63	16	86	74	57	862	12	110
Median	50	17	1.6	24	15	15	39	1.9	15
75th percentile	68	27	2.7	33	24	24	119	2.8	23
Mean	56	20	2.4	25	18	18	101	2.5	21
SD	37	13	2.6	18	14	13	160	2.5	21

Abbreviations: mCPP, mono(3-carboxypropyl) phthalate; mMP, monomethyl phthalate. Values < LOD were set to LOD/2.

**Table 6:** Comparison of urinary concentrations (ng/mL) by percentile in the present study (Sweden), a U.S. study on ADG, the 1999–2000 NHANES, and a German study.

	Sweden		USA ADG^a^		USA NHANES^b^		Germany^c^
Metabolite	50th	75th	50th	75th	50th	75th	50th
Monoester							
mBP^d^	45.5	68	13.5	30.9	30.0	59.5	181
mBzP^d^	13.2	20	8.3	23.5	16.0	35.8	21.0
mCPP	1.5	2.5	2.1	3.6			
mEP^d^	35	80	128.4	437	174	425	90.2
miBP^d^	16.4	30	2.5	5.1			
mMP	1.2	2.8	0.7	3.2			
DEHP							
mEHHP	14.6	29	11.4	20.1			46.8
mEHP	9.0	17	3.3	9.0	3.00	7.00	10.3
mEOHP	11.3	24	11.1	19.0			36.5

Abbreviations: mCPP, mono(3-carboxypropyl) phthalate; mMP, monomethyl phthalate.

a Swan et al. (2005).

b Silva et al. (2004a).

c Koch et al. (2003b).

d Correlated with AGD (Swan et al. 2005).
